# Incidence of Monkeypox Among Unvaccinated Persons Compared with Persons Receiving ≥1 JYNNEOS Vaccine Dose — 32 U.S. Jurisdictions, July 31–September 3, 2022

**DOI:** 10.15585/mmwr.mm7140e3

**Published:** 2022-10-07

**Authors:** Amanda B. Payne, Logan C. Ray, Kiersten J. Kugeler, Amy Fothergill, Elizabeth B. White, Michelle Canning, Jennifer L. Farrar, Leora R. Feldstein, Adi V. Gundlapalli, Kennedy Houck, Jennifer L. Kriss, Nathaniel M. Lewis, Emily Sims, Dawn K. Smith, Ian H. Spicknall, Yoshinori Nakazawa, Inger K. Damon, Amanda C. Cohn, Daniel C. Payne

**Affiliations:** ^1^CDC Monkeypox Emergency Response Team; ^2^Epidemic Intelligence Service, CDC.

Human monkeypox is caused by *Monkeypox virus* (MPXV), an Orthopoxvirus, previously rare in the United States (*1*). The first U.S. case of monkeypox during the current outbreak was identified on May 17, 2022 (*2*). As of September 28, 2022, a total of 25,341 monkeypox cases have been reported in the United States.* The outbreak has disproportionately affected gay, bisexual, and other men who have sex with men (MSM) (*3*). JYNNEOS vaccine (Modified Vaccinia Ankara vaccine, Bavarian Nordic), administered subcutaneously as a 2-dose (0.5 mL per dose) series with doses administered 4 weeks apart, was approved by the Food and Drug Administration (FDA) in 2019 to prevent smallpox and monkeypox infection (*4*). U.S. distribution of JYNNEOS vaccine as postexposure prophylaxis (PEP) for persons with known exposures to MPXV began in May 2022. A U.S. national vaccination strategy^†^ for expanded PEP, announced on June 28, 2022, recommended subcutaneous vaccination of persons with known or presumed exposure to MPXV, broadening vaccination eligibility. FDA emergency use authorization (EUA) of intradermal administration of 0.1 mL of JYNNEOS on August 9, 2022, increased vaccine supply (*5*). As of September 28, 2022, most vaccine has been administered as PEP or expanded PEP. Because of the limited amount of time that has elapsed since administration of initial vaccine doses, as of September 28, 2022, relatively few persons in the current outbreak have completed the recommended 2-dose series.^§^ To examine the incidence of monkeypox among persons who were unvaccinated and those who had received ≥1 JYNNEOS vaccine dose, 5,402 reported monkeypox cases occurring among males^¶^ aged 18–49 years during July 31–September 3, 2022, were analyzed by vaccination status across 32 U.S. jurisdictions.** Average monkeypox incidence (cases per 100,000) among unvaccinated persons was 14.3 (95% CI = 5.0–41.0) times that among persons who received 1 dose of JYNNEOS vaccine ≥14 days earlier. Monitoring monkeypox incidence by vaccination status in timely surveillance data might provide early indications of vaccine-related protection that can be confirmed through other well-controlled vaccine effectiveness studies. This early finding suggests that a single dose of JYNNEOS vaccine provides some protection against monkeypox infection. The degree and durability of such protection is unknown, and it is recommended that people who are eligible for monkeypox vaccination receive the complete 2-dose series.

Aggregate weekly numbers of confirmed and probable monkeypox cases^††^ among males aged 18–49 years with illness onset^§§^ during July 31–September 3, 2022, were analyzed across 32 public health jurisdictions. These jurisdictions routinely ascertain vaccination status^¶¶^ through patient interview or link cases with vaccination data from their immunization registries and separately submit deidentified vaccine administration data to CDC. The analysis was limited to males aged 18–49 years to exclude persons who might have received routine smallpox vaccination in childhood. Persons with monkeypox were categorized as 1) unvaccinated; 2) potentially vaccinated, without date of vaccination; 3) vaccinated, with illness onset ≤13 days after their first dose; or 4) vaccinated, with illness onset ≥14 days after their first dose.***

Vaccination coverage was estimated as the total number of persons vaccinated as of 2 weeks before the start date of a week, divided by the estimated population eligible for vaccination.^†††^ This underlying population included persons in each jurisdiction who might benefit from expanded vaccination in the context of the outbreak and was estimated as the number of MSM with HIV or who are eligible for HIV preexposure prophylaxis (HIV-PrEP) (*6*). The number of eligible unvaccinated persons was obtained by subtracting the number of vaccinated persons from estimates of the vaccine-eligible population. Weekly^§§§^ incidence by vaccination status was calculated as the number of cases divided by the number of persons either unvaccinated as of that week or vaccinated as of 2 weeks earlier.^¶¶¶^ Because relatively few persons had received a second vaccine dose within the time frame of this analysis, incidence among persons who had received their first JYNNEOS vaccine dose ≥14 days earlier is reported. Persons with illness onset ≤13 days after receipt of their first dose of vaccine, potentially vaccinated persons (those without a documented date of vaccination), and persons vaccinated before 2022 were excluded from the analysis. The average incidence rate ratio (IRR) during the study period was calculated by dividing the weighted average incidence across all weeks among unvaccinated persons by that among vaccinated persons; a 95% CI for the average IRR was calculated to account for variation in weekly rates. Weighting was based on the population size in each vaccination status category.

Two sensitivity analyses were conducted. The first examined changes in IRR when considering the total estimated MSM population as eligible for vaccination. The second examined changes in IRR under the assumptions that 50% or 100% of persons with monkeypox with unknown vaccination date received vaccine ≥14 days before illness onset. SAS (version 9.4; SAS Institute) and R (version 4.0.3; R Foundation) were used to conduct all analyses. This activity was reviewed by CDC and was conducted consistent with applicable federal law and CDC policy.****

During July 31–September 3, 2022, among 32 jurisdictions reporting 6,471 monkeypox cases (range across jurisdictions = 2–2,186 cases), a total of 5,402 (83.5%) were reported among males aged 18–49 years (Table). Among these, a total of 4,606 (85.3%) cases were among unvaccinated persons, 269 (5.0%) were among persons whose illness onset occurred ≤13 days after receipt of their first vaccine dose, 77 (1.4%) were among persons with illness onset ≥14 days after receipt of their first vaccine dose, and 450 (8.3%) were among persons without a known vaccination date. No persons vaccinated before 2022 were identified. Population coverage with 1 vaccine dose as of 2 weeks before the start of each week increased from 5.2% (July 31) to 29.9% (August 28) in the 32 jurisdictions; coverage with two vaccine doses increased from 0.1% to 1.9%. As of September 23, 2022, 10 and 2 cases had been reported in persons who had received a second JYNNEOS vaccine ≤13 days and ≥14 days before illness onset, respectively.

**TABLE:** JYNNEOS vaccination coverage among males* aged 18–49 years and monkeypox cases by first-dose vaccination status^†^ — 32 U.S. jurisdictions,^§,^^¶^ July 31–September 3, 2022

Characteristic	No. (%), by week beginning					Total
	Jul 31	Aug 7	Aug 14	Aug 21	Aug 28	
1-dose vaccination coverage, %**	5.2	9.8	16.2	23.9	29.9	**NA**
**2-dose vaccination coverage,%^††^**	0.1	0.2	0.3	0.8	1.9	**NA**
**Total monkeypox cases^§§^**	**1,284**	**1,313**	**1,034**	**1,013**	**758**	**5,402**
**Vaccination status**						
Unvaccinated	1,097 (85.4)	1,103 (84.0)	872 (84.3)	881 (87.0)	653 (86.1)	**4,606 (85.3)**
Vaccinated	187 (14.6)	210 (16.0)	162 (15.7)	132 (13.0)	105 (13.9)	**796 (14.7)**
**Vaccination date known**						
No	121 (9.4)	118 (9.0)	79 (7.6)	78 (7.7)	54 (7.1)	**450 (8.3)**
Yes	66 (5.1)	92 (7.0)	83 (8.0)	54 (5.3)	51 (6.7)	**346 (6.4)**
**Illness onset relative to vaccination (among those with known vaccination date)**						
0–13 days after first dose	62 (4.8)	73 (5.6)	65 (6.3)	39 (3.8)	30 (4.0)	**269 (5.0)**
≥14 days after first dose	4 (0.3)	19 (1.4)	18 (1.7)	15 (1.5)	21 (2.8)	**77 (1.4)**
Before second dose	4 (0.3)	17 (1.3)	16 (1.5)	11 (1.1)	17 (2.2)	**65 (1.2)**
0–13 days after second dose	0 (—)	2 (0.2)	1 (0.1)	4 (0.4)	3 (0.4)	**10 (0.2)**
≥14 days after second dose	0 (—)	0 (—)	1 (0.1)	0 (—)	1 (0.1)	**2 (0.1)**

**Abbreviations**: NA = not applicable; PCR = polymerase chain reaction.

* Defined as sex assigned at birth or gender identity.

^†^ Vaccinated: persons who had received ≥ 1 dose of JYNNEOS vaccine.

^§^ Alaska, California, Colorado, Georgia, Hawaii, Idaho, Illinois, Iowa, Kansas, Kentucky, Louisiana, Maine, Maryland, Massachusetts, Michigan, Missouri, Montana, Nevada, New Hampshire, New Mexico, North Dakota, Oklahoma, Oregon, Pennsylvania, Rhode Island, South Carolina, South Dakota, Tennessee, Utah, Virginia, West Virginia, and Wisconsin.

^¶^ Jurisdictions were included if age and sex assigned at birth or gender identity were available for ≥70% of cases reported, vaccination status was available for ≥50% of cases in males (defined by either sex assigned at birth or gender identity) aged 18–49 years or the jurisdiction confirmed that cases are linked to immunization registry entries, and de-identified vaccination administration data were submitted to CDC.

** Proportion of population eligible for vaccination that had received 1 dose of JYNNEOS vaccine as of 2 weeks before the start of the week.

^††^ Proportion of population eligible for vaccination that had received 2 doses of JYNNEOS vaccine as of 2 weeks before the start of the week. ^§§^ Confirmed (presence of *Monkeypox virus* DNA by PCR testing or Next-Generation sequencing of a clinical specimen or isolation of *Monkeypox virus* in culture from a clinical specimen) and probable (presence of *Orthopoxvirus* DNA by PCR testing, or *Orthopoxvirus* using immunohistochemical or electron microscopy or detectable levels of anti-*Orthopoxvirus* immunoglobulin M antibody) monkeypox cases.

Weekly monkeypox incidence during July 31–September 3 was higher among unvaccinated persons than among those who had received their first JYNNEOS vaccine dose ≥14 days earlier (Figure). Average IRR comparing unvaccinated persons with those who received 1 dose of vaccine ≥14 days earlier was 14.3 (95% CI = 5.0–41.0). A sensitivity analysis expanding the estimated number of persons eligible for vaccination yielded similar trends but lower average IRR (Supplementary Figure, https://stacks.cdc.gov/view/cdc/121578). A sensitivity analysis examining changes to IRR assuming 50% or 100% of persons with unknown vaccination date received their vaccine dose ≥14 days before illness onset yielded similar trends but lower average IRR (Supplementary Table, https://stacks.cdc.gov/view/cdc/121579).

**FIGURE:**
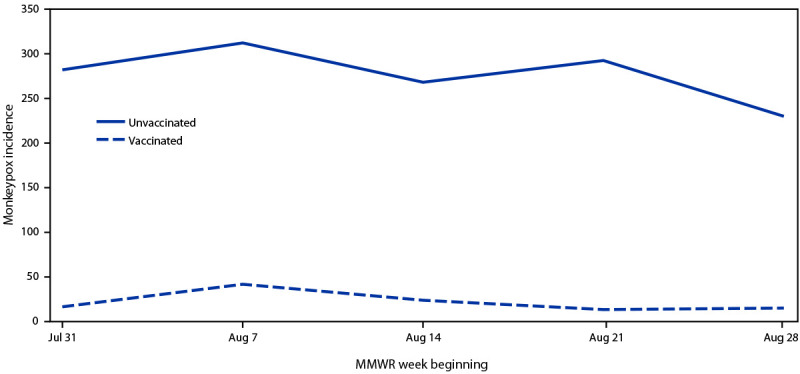
Weekly monkeypox incidence,* by first-dose vaccination status^†,§^ among males aged 18–49 years eligible for vaccination^¶^ — 32 U.S. jurisdictions**^,^ ^††^ July 31–September 3, 2022 **Abbreviation**: IRR = incidence rate ratio. * Cases per 100,000 population. Rate in vaccinated persons = number of probable or confirmed cases reported to CDC with date of illness onset, specimen collection, lab test completion, admission, diagnosis, discharge, case investigation start date, or date first electronically submitted or reported to the county, state, or public health department (earliest available date) ≥14 days after receiving the first dose of JYNNEOS vaccine among total vaccinated population as of 2 weeks previously. Rate in unvaccinated persons = number of probable or confirmed cases reported to CDC without evidence of vaccination among total unvaccinated population. † Vaccinated = persons who had received ≥1 dose of JYNNEOS ≥14 days earlier. ^§^ Average IRR comparing unvaccinated persons with those who received 1 dose of vaccine ≥14 days earlier was 14.3. ^¶^ Gay, bisexual, and other men who have sex with men who have HIV infection or who are eligible to receive HIV preexposure prophylaxis were considered eligible for vaccination. ** Alaska, California, Colorado, Georgia, Hawaii, Idaho, Illinois, Iowa, Kansas, Kentucky, Louisiana, Maine, Maryland, Massachusetts, Michigan, Missouri, Montana, Nevada, New Hampshire, New Mexico, North Dakota, Oklahoma, Oregon, Pennsylvania, Rhode Island, South Carolina, South Dakota, Tennessee, Utah, Virginia, West Virginia, and Wisconsin. †† Jurisdictions were included if age and sex assigned at birth or gender identity was available for ≥70% of cases reported, vaccination status was available for ≥50% of cases in males (defined by either sex assigned at birth or gender identity) aged 18–49 years or the jurisdiction confirmed cases were linked to immunization registry entries, and de-identified vaccination administration data were submitted to CDC.

## Discussion

Among 32 U.S. jurisdictions, monkeypox incidence among persons who were currently recommended to receive PEP or expanded PEP with JYNNEOS vaccine was higher among unvaccinated persons compared with those who had received their first vaccine dose ≥14 days earlier. Data for this analysis were collected during a period when vaccine was widely available, reducing potential bias from limited vaccine accessibility. Findings are consistent with recent studies reporting that a single dose of JYNNEOS vaccine for prevention of MPXV infection in males aged 18–42 years who were prescribed HIV-PrEP or with diagnosed HIV infection and one or more other sexually transmitted infection might provide some protection (*7*) and modest induction of antibody levels after a single dose (*8*).

The findings in this report are subject to at least six limitations. First, linkage of monkeypox case surveillance and vaccination administration data might result in misclassifications that could influence IRR estimates. Some patients might not be linkable within a jurisdiction’s immunization registry because of receipt of vaccine outside the jurisdiction, or interviewed persons with monkeypox might have incorrectly reported their own vaccination status. This approach assumes that persons with unknown vaccination status were unvaccinated and excludes those with unknown date of vaccination because timing between vaccination and illness onset could not be established. Second, this analysis was unable to control for possible differences in testing or behaviors that increase risk for MPXV exposure or possible differences in risk because of patient characteristics (e.g., age and underlying medical conditions, including HIV status); consequently, causality and a full attribution of these results to vaccination cannot be inferred from these data. Third, incidence among persons who received 2 JYNNEOS vaccine doses could not be assessed, because of low second dose coverage and sparse data during the study period precluded these estimates. Fourth, temporality of exposures causing infection are not known. Vaccination strategies focused on PEP and expanded PEP during the study period; however, some patients might have received vaccine before exposure, or might have had additional exposures after vaccination. Fifth, confirmation that all identified persons with monkeypox were members of the population eligible for vaccination was not possible. Finally, data assessed from 32 jurisdictions accounted for 56% of the U.S. population eligible for vaccination and might not be generalizable.

These data are intended to provide an early indication of the real-world impact of vaccination with JYNNEOS for preventing monkeypox and to guide public health prevention interventions (e.g., vaccinating persons at high risk for infection while still encouraging harm reduction strategies, including reducing the number of sexual partners and one-time sexual encounters) (*9*). The framework used in this analysis allows for ongoing comparison of observed IRRs over time and can be used to monitor vaccine performance after a second dose. Durability of immunity after a single dose is not yet known, and because vaccine effectiveness and duration of protection are anticipated to be better after 2 doses, it remains important that all vaccinated persons receive their second dose. Monitoring monkeypox incidence by vaccination status using currently available surveillance data might provide early estimates of vaccine performance for rapid public health decision making. Although the findings are encouraging, corroboration and confirmation through planned epidemiologic studies that are better able to account for potential biases are needed. This early finding suggests that a single dose of JYNNEOS vaccine provides some protection against monkeypox infection. It is recommended people who are eligible for monkeypox vaccination receive the complete 2-dose series.

**Summary**

**What is already known about this topic?**

Real-world monkeypox vaccine performance data are limited in the context of the ongoing monkeypox outbreak.

**What is added by this report?**

Across 32 U.S. jurisdictions, among males aged 18–49 years eligible for JYNNEOS vaccination, monkeypox incidence was 14 times as high among unvaccinated males compared with those who had received a first vaccine dose ≥14 days earlier.

**What are the implications for public health practice?**

These early findings suggest that a single JYNNEOS dose provides some protection against monkeypox infection. The degree and durability of such protection is unknown, and it is recommended that persons who are eligible for monkeypox vaccination receive the complete 2-dose series.

## Footnotes

* https://www.cdc.gov/poxvirus/monkeypox/response/2022/us-map.html

† https://www.hhs.gov/about/news/2022/06/28/hhs-announces-enhanced-strategy-vaccinate-protect-at-risk-individuals-from-current-monkeypox-outbreak.html

§
https://www.cdc.gov/poxvirus/monkeypox/response/2022/vaccines_data.html

¶ Cases reflect infections occurring among persons who self-reported sex assigned at birth or self-reported gender identity as male.

** Alaska, California, Colorado, Georgia, Hawaii, Idaho, Illinois, Iowa, Kansas, Kentucky, Louisiana, Maine, Maryland, Massachusetts, Michigan, Missouri, Montana, Nevada, New Hampshire, New Mexico, North Dakota, Oklahoma, Oregon, Pennsylvania, Rhode Island, South Carolina, South Dakota, Tennessee, Utah, Virginia, West Virginia, and Wisconsin. Jurisdictions were included if age and sex assigned at birth or gender identity was available for ≥70% of cases reported, vaccination status was available for ≥50% of cases in males (defined by either sex assigned at birth or gender identity) aged 18–49 years or the jurisdiction confirmed cases are linked to immunization registry entries, and de-identified vaccination administration data were submitted to CDC.

†† Confirmed (presence of *Monkeypox virus* DNA by polymerase chain reaction [PCR] testing or Next-Generation sequencing of a clinical specimen OR isolation of *Monkeypox virus* in culture from a clinical specimen) and probable (presence of *Orthopoxvirus* DNA by PCR, or *Orthopoxvirus* using immunohistochemical or electron microscopy or detectable levels of anti-*Orthopoxvirus* immunoglobulin M antibody) monkeypox cases.

§§ Illness onset date refers to the earliest date available for each case. Dates available for selection varied by how the case was reported to the system and include illness onset, specimen collection, lab test completion, admission, diagnosis, discharge, case investigation start date, or date first electronically submitted or reported to the county, state, or public health department.

¶¶ Receipt of ≥1 dose of JYNNEOS vaccine.

*** Unvaccinated: No evidence in case record of receipt of JYNNEOS vaccine or vaccination date after illness onset, including records for which vaccination information was unknown. Potentially vaccinated: case record reflected some indication of vaccination, but without dose number or date. Vaccinated, illness onset ≤13 days after first dose: illness onset ≤13 days of receiving first dose of JYNNEOS vaccine. Vaccinated, illness onset ≥14 days after first dose: illness onset ≥14 days after receiving first dose of JYNNEOS vaccine, excluding persons vaccinated for smallpox before 2022.

††† The population aged 18–49 years that might benefit from expanded vaccination includes MSM with HIV infection (jurisdiction-specific estimates of 2020 HIV prevalence are from CDC’s Atlas Plus [https://www.cdc.gov/nchhstp/atlas/index.htm] describing MSM who acquired HIV through male-to-male sexual contact or male-to-male sexual contact and injection drug use) or who are eligible for HIV-PrEP (estimated as the ratio of the jurisdiction-specific number of MSM receiving HIV preexposure prophylaxis (HIV-PrEP) and the jurisdiction-specific HIV-PrEP coverage. The number of MSM with HIV or who are eligible for HIV-PrEP aged 18–49 years was estimated by aggregating 2021 U.S. Census Bureau estimates for males aged 0–12, 13–17, 18–49, and ≥50 years, calculating the state proportion in each age group, and multiplying by the estimated number of MSM with HIV or who are eligible for HIV-PrEP in each state to obtain proportional distributions. Additional details about these methods can be obtained by contacting the corresponding author.

§§§ Cases and vaccine doses administered were aggregated by MMWR week. Weeks begin on Sunday and end on Saturday.

¶¶¶ Because most vaccine administered during the study period was PEP, this time point was chosen to account for the incubation period after exposure. Further, immunogenicity data submitted to FDA indicated that antibody titers 2 weeks after the first dose were similar to titers 4 weeks after the first dose and were significantly higher than prevaccination antibody titers.

**** 5 C.F.R. part 46, 21 C.F.R. part 56; 42 U.S.C. Sect. 241(d); 5 U.S.C. Sect. 552a; 44 U.S.C. Sect. 3501 et seq.
